# Association of a Serum Uric Acid-Related Dietary Pattern with Metabolic Syndrome Among Guangzhou Children Aged 9–17 Years: A Cross-Sectional Study

**DOI:** 10.3390/nu17162618

**Published:** 2025-08-13

**Authors:** Wanzhen Zhong, Shiyun Luo, Guixian Tao, Jiayi Wan, Jinhan Fu, Cunzi Zeng, Jie Huang, Xi Chen, Nali Deng, Weiwei Zhang, Jing Gu, Yan Li

**Affiliations:** 1 School of Public Health, Sun Yat-Sen University, Guangzhou 510080, China; zhongwzh6@mail2.sysu.edu.cn (W.Z.); taogx@mail2.sysu.edu.cn (G.T.); wanjy26@mail2.sysu.edu.cn (J.W.); fujh6@mail2.sysu.edu.cn (J.F.); gujing5@mail.sysu.edu.cn (J.G.); 2 Guangzhou Center for Disease Control and Prevention, Guangzhou Health Supervision Institute, Guangzhou 510440, China; luoshy25@mail3.sysu.edu.cn (S.L.); gzcdc_zengcz@gz.gov.cn (C.Z.); huangjie1026@126.com (J.H.); gzcdc_chenx@gz.gov.cn (X.C.); gzcdczhangww@foxmail.com (W.Z.); 3 Guangzhou Health Care Promotion Center for Primary and Middle Schools, Guangzhou 510180, China; nali2000@126.com

**Keywords:** metabolic syndrome, dietary pattern, serum uric acid, reduced-rank regression, children

## Abstract

**Background:** Childhood metabolic syndrome (MetS) represents a growing public health concern in China, with diet emerging as a critical modifiable risk factor. Although numerous studies have explored the relationship between dietary patterns and MetS, the specific influence of dietary patterns associated with serum uric acid (SUA) levels in the young population remains poorly understood. The purpose of this study is to investigate the relationship between an SUA-related dietary pattern and MetS risk among children aged 9–17 years in Guangzhou, China. **Methods**: A cross-sectional study was conducted in Guangzhou from March 2023 to May 2024, including 4181 participants. To identify the dietary pattern associated with SUA, we employed reduced-rank regression (RRR) using 15 predefined food groups. The multivariate logistic regression models were applied to evaluate the association between the SUA-related dietary pattern scores (as continuous variables and tertiles) and the risk of MetS and its five components. **Results**: The SUA-related dietary pattern was characterized by high consumption of meat & meat products and beverages, and low consumption of fresh vegetables, fresh fruits & their products, eggs, dairy products, and sugary food. After adjusting for confounding factors, each one-unit increase in the SUA-related dietary pattern score corresponded to 27% higher odds of MetS (OR = 1.27, 95% CI: 1.00–1.62), and 24% higher odds of central obesity (OR = 1.24, 95% CI: 1.11–1.38). In subgroup analyses, higher adherence to the SUA-related dietary pattern scores was linked to significantly increased odds of MetS (adjusted OR = 1.69, 95% CI: 1.18–2.42) and central obesity (adjusted OR = 1.39, 95% CI: 1.20–1.62) among participants with insufficient physical activity. **Conclusions:** Higher adherence to the SUA-related dietary pattern was associated with higher odds of MetS in Chinese children, particularly among those with insufficient physical activity. The results provide new insights into the relationship between dietary patterns and childhood MetS, offering dietary strategies focused on managing SUA levels to prevent MetS.

## 1. Introduction

Metabolic syndrome (MetS) is a cluster of cardiovascular and metabolic risk factors, including central obesity, elevated blood pressure, hyperglycemia and dyslipidemia. It has become a significant public health issue among children [1,2]. Epidemiological studies show that the prevalence of childhood MetS is rising in parallel with the global increase in childhood obesity [3,4]. In China, the overall MetS prevalence among children (7–17 years) increased from 3.7% in 2002 to 5.98% by 2016–2017 [5,6]. Moreover, childhood MetS is associated with elevated inflammatory markers, increased carotid intima-media thickness, and a higher risk of developing non-alcoholic fatty liver disease, all of which contribute to increased risk of cardiovascular disease risk in adulthood [7,8,9,10]. Hence, identifying modifiable factors influencing childhood MetS is crucial for developing targeted prevention strategies.

Diet habits are a key modifiable factor strongly associated with the occurrence and development of MetS [11]. Dietary pattern analysis offers a more biologically relevant approach than single-nutrient investigations. It accounts for synergistic and antagonistic interactions among food groups, thereby better elucidating the complex relationship between diet and MetS [12]. However, findings on dietary patterns and childhood MetS remain inconsistent. For example, some studies have shown a negative correlation between the Dietary Approaches to Stop Hypertension (DASH) diet and MetS in American adolescents [13], while similar associations were not observed in Iranian adolescents [14]. These studies usually use traditional methods such as hypothesis-driven and data-driven approaches to identify dietary patterns. On the one hand, hypothesis-driven methods, such as the DASH diet, rely on predefined criteria but struggle to capture the real-world food–nutrient interactions [15]. On the other hand, data-driven methods, like factor analysis, extract dietary patterns from data but often lack a biological basis, making it hard to clarify their link with diseases [16]. Given these limitations, there is a pressing need to find better ways to derive dietary patterns.

Reduced-rank regression (RRR) provides a novel method for assessing dietary patterns, combining the strengths of both hypothesis-driven and data-driven approaches [17]. It clarifies how specific dietary components affect disease via biological pathways [18,19,20]. In practice, RRR constructs linear combinations from dietary predictors (specific foods/food groups) based on a priori hypotheses (e.g., MetS), to maximize the explained variance in response variables (e.g., disease-specific biomarkers) [21]. Selecting an appropriate response variable is critical in RRR, as it affects the accuracy and relevance of the identified dietary patterns. For childhood MetS biomarkers, serum uric acid (SUA) is notable. It has been shown to independently predict the occurrence of MetS in adults [22] and children [23]. Moreover, the Lingnan diet in Guangzhou, which is rich in high-purine foods like seafood and preserved meats [24], may elevate SUA levels above the national average [25]. Thus, SUA is an ideal response variable for deriving Guangzhou-specific dietary patterns via RRR.

Therefore, this study aims to use RRR to identify a dietary pattern related to SUA levels and explore its connections with MetS and its components among children aged 9–17 in Guangzhou. The findings will provide scientific evidence to inform nutritional education and intervention strategies for childhood MetS in Guangzhou.

## 2. Materials and Methods

### 2.1. Study Population

The dataset for this study was obtained from the Nutrition and Health Surveillance for Students in Guangzhou. A cross-sectional study was carried out in Guangzhou from March 2023 to May 2024. Participants were selected using a cluster random sampling, as described in previous articles by this research group [26].

The minimum sample size was calculated using this formula:$\;N=deff\frac{Z_{\alpha /2}^{2}\times p(1-p)}{\delta^{2}}$ [27]. Parameters were set as follows: a two-sided confidence level of 95% ($Z_{\alpha /2}=1.96);$ a prevalence rate (*p*) of 4.0% for MetS among Chinese children [28]; the design effect (*deff*) was set to 1.4 [29]; and the relative error (*r*) was 20%, which resulted in δ = 20% × 4.0%. Given these parameter settings, the initial calculation suggested that a baseline sample size of 3227 participants would be required. To address potential attrition due to invalid questionnaires and anticipated non-participation rates, a 10% upward adjustment was applied to ensure methodological robustness and data reliability. Consequently, the final required sample size was determined to be at least 3586 student participants, thereby maintaining statistical power and safeguarding the validity of study outcomes.

**Figure 1:**
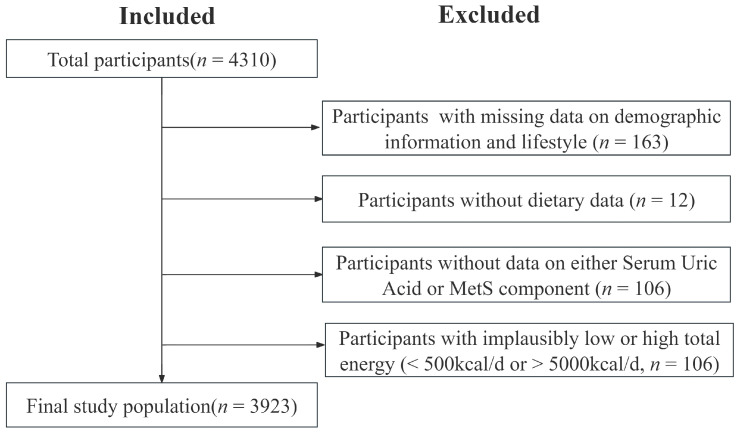
Flow diagram of study population.

After excluding participants with incomplete data on key variables, the final sample included 3923 participants (Figure 1). The study protocol was approved by the Institutional Review Board of Guangzhou Center for Disease Control and Prevention (ethics number: GZCDC-ECHR-2022P0038). Written informed consent was obtained from all participants and their respective parents or guardians for participation in this research.

### 2.2. Data Collection

Data were collected from children through face-to-face interviews conducted by research assistants who had undergone standardized training. The survey included questionnaires, physical examinations, and laboratory assessments.

(1) Questionnaires: (1) Demographics: age, sex, the education level of father and mother. (2) Lifestyle: boarding status, insufficient physical activity (according to the Physical Activity Guidelines for Chinese(2021) [30,31], defined as moderate-to-vigorous physical activity lasting less than 1 h per day and outdoor activity lasting less than 2 h per day), screen time, and sleep duration. (3) Dietary assessment: To evaluate the frequency and quantity of food consumption among children over the past month, a semi-quantitative food frequency questionnaire (FFQ) was employed. This FFQ was derived from the instrument used in the 2015 China National Chronic Non-Communicable Disease and Nutrition Surveillance [32], and subsequently adapted by a team of specialists in epidemiology and nutrition to better align with the dietary patterns of children in Guangzhou. Prior research verified that this FFQ possesses satisfactory reproducibility and validity [33], and it has shown strong consistency in dietary surveys carried out in Guangzhou [34]. Grounded in the Chinese Food Composition Table Standard Edition (6th edition) [35], the FFQ includes 66 types of food spanning 15 categories (Table S1). Considering the potential difficulties that children may encounter when reporting food information, the study simultaneously provided food maps and food models as auxiliary tools to further improve the accuracy of their reported food intake, thereby helping children describe their food intake more clearly.

(2) Physical measurements: Trained surveyors used uniformly procured equipment to measure the respondents’ height, weight, blood pressure, and waist circumference. All measurement devices and methods complied with the National Health Monitoring Standards of China: Anthropometric measurements method in health surveillance [36].

(3) Laboratory assessments: Blood biochemical parameters included fasting plasma glucose (FPG), serum total cholesterol (TC), triglycerides (TG), high-density lipoprotein cholesterol (HDL-C), and serum uric acid (SUA). FPG was assessed using the hexokinase method. TC, TG, and SUA were quantified via enzymatic oxidase methods. HDL-C levels were determined using direct homogeneous assay techniques. Non-high-density lipoprotein cholesterol (non-HDL-C) was calculated by subtracting HDL-C from TC (non-HDL-C = TC − HDL-C).

### 2.3. Definition of MetS

In the study, MetS was diagnosed based on the criteria proposed by the Chinese Expert Consensus on the Definition of Metabolic Syndrome and Prophylaxis and Treatment Proposal in Children and Adolescents [37].

For children aged ≥ 10 years, the diagnostic criteria for MetS included the presence of central obesity (waist circumference ≥ the 90th percentile [P_90_] for age and sex [38,39]) plus at least two of the following abnormalities: elevated blood pressure (systolic or diastolic blood pressure ≥ the 95th percentile [P_95_] for age and sex [40]), hyperglycemia (FBG ≥ 5.6 mmol/L), hypertriglyceridemia (TG ≥ 1.47 mmol/L), or dyslipidemia (HDL-C < 1.03 mmol/L or non-HDL-C ≥ 3.76 mmol/L).

For children aged under 10 years old, the diagnostic threshold for central obesity was adjusted to waist circumference ≥ P_95_ for age and sex [39]. This adjustment followed literature recommendations and was made while other diagnostic cutoffs remained unchanged. The modified MetS diagnostic approach for children under 10 has been adopted in multiple prior studies [41,42].

### 2.4. SUA-Related Dietary Pattern

In the current study, 15 food groups were used as predictive variables, with their intakes adjusted for energy intake using the following formula: $Adjusted\;food\;groups\;intake\;(g/1000\;kcal)=\frac{Daily\;food\;groups\;intake\;(g/d)}{Total\;daily\;energy\;intake(kcal/d)}\times 1000$ [43]. Meanwhile, the log-transformed SUA served as the response variable in the RRR analysis. Specifically, the derived dietary pattern explained the maximal variation in the log-transformed SUA and was expressed as an optimized linear combination of these food groups. Here, factor loadings indicated the relative contributions of each group, and food groups with factor loadings having absolute values of >0.2 were deemed to be significant contributors [44]. Individual dietary pattern scores were calculated by summing z-standardized food intake values, weighted by their factor loadings. This method is based on the eigen decomposition of the covariance matrix between predictors and response variables [17,21].

### 2.5. Statistical Analyses

The following analytical approaches were employed in this study: Quantitative variables were described using medians with interquartile ranges [*M* (*P*_25_, *P*_75_)] and compared between groups via Mann–Whitney U tests or the Kruskal–Wallis test, while categorical variables were presented as rates or proportions and analyzed using the chi-square trend test.

To assess associations between the SUA-related dietary pattern scores and MetS components, the dietary scores were categorized into tertiles (T1: lowest, T2: middle, T3: highest) for multivariate logistic regression analyses. T1 was designated as the reference category to estimate odds ratios (ORs) and 95% confidence intervals (CIs) for T2 and T3. Subsequently, the same regression models were applied to analyze dietary scores as continuous variables, enabling the assessment of dose–response relationships by estimating the *OR* for each 1-unit increment in the score. Two models were constructed: Model 1 (unadjusted) and Model 2 (adjusted for age, sex, boarding status, physical activity, screen time, sleep duration, and the education level of father and mother). Furthermore, given the small number of MetS cases (<100), the Firth correction was applied in analyses with MetS as the outcome [45]. Additionally, subgroup analyses were conducted to examine the relationship between SUA-related dietary pattern scores and MetS components by physical activity level (insufficient vs. sufficient) [46].

Dietary pattern derivation was implemented in SAS^®^ Studio version 9.4, while subsequent statistical procedures were executed using R 4.4.1. A two-tailed significance threshold of *p* < 0.05 was uniformly applied across all analytical stages. GraphPad Prism 10.1 and Microsoft Office 2021 were used for graphs.

## 3. Results

### 3.1. Demographic and Basic Characteristics of the Study Population

The general characteristics of the study population are presented according to their MetS statuses in Table 1. The general characteristics of the study population are also presented based on the status of individual MetS components in Table 2. Among the 3923 children in the study, 86 cases of MetS were identified, representing a prevalence of 2.20%. Compared to the normal group, MetS cases had a higher proportion of males and exhibited elevated waist circumference, systolic/diastolic blood pressure, FBG, TG, non-HDL-C, and SUA levels, along with lower HDL-C levels (all *p* < 0.001).

### 3.2. SUA-Related Dietary Pattern and Its Characteristics

**Figure 2:**
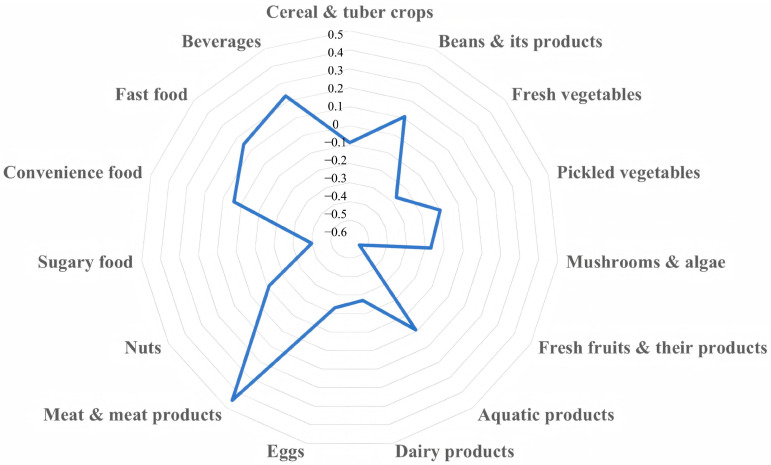
Factor loadings of 15 food groups by RRR analysis.

As shown in Figure 2 and Table S2, the SUA-related dietary pattern was characterized by high consumption of meat & meat products and beverages, and low consumption of fresh vegetables, fresh fruits & their products, eggs, dairy products, and sugary food.

As indicated in Table 3, individuals characterized by higher SUA-related dietary pattern scores were more likely to be older, male, and boarding students, and to have a higher prevalence of longer screen time and shorter sleep duration (*p* < 0.001).

### 3.3. Relationship Between the SUA-Related Dietary Pattern Scores and MetS

#### 3.3.1. Analysis of the SUA-Related Dietary Pattern Scores and MetS

After adjusting for confounders, a significant positive linear association was observed between the SUA-related dietary pattern scores and MetS (adjusted OR = 1.27, 95% CI: 1.00–1.62, *p* = 0.049). The association exhibited component-specific variations when analyzing individual MetS diagnostic criteria. Specifically, each one-unit increase in the SUA-related dietary score demonstrated was associated with the presence of central obesity, with an adjusted OR of 1.24 (adjusted OR = 1.24, 95% CI: 1.11–1.38, *p* < 0.001). (Table 4).

Further comparative analyses indicated that participants in the highest tertile (T3) of the SUA-related dietary pattern scores had a higher likelihood of central obesity than those in the lowest tertile (T1) (adjusted OR_T3vs.T1_ = 1.72, 95% CI: 1.33–2.25, *p* < 0.001). However, no statistically significant associations were detected between the SUA-related dietary scores and other MetS components (all *p* > 0.05). (Table 4).

#### 3.3.2. Analysis of the SUA-Related Dietary Pattern Scores and Childhood MetS in Children of Different Physical Activity Sufficiency

Table 5 presents the subgroup analysis results. Within the insufficient physical activity group, higher adherence to the SUA-related dietary pattern score was associated with significantly increased odds of MetS (adjusted OR = 1.69, 95% CI: 1.18–2.42, *p* = 0.004) and central obesity (adjusted OR = 1.39, 95% CI: 1.20–1.62, *p* < 0.001). Similar findings were observed in the detailed tertile analyses.

## 4. Discussion

In this study, we identified a dietary pattern associated with SUA using RRR among children aged 9–17 years in Guangzhou. We further investigated the associations of this dietary pattern with MetS and its individual components. Results showed that for each one-unit increase in the SUA-related dietary pattern score, the risk of developing MetS and central obesity increased by 27% and 24%, respectively. In subgroup analyses, higher adherence to this dietary pattern was linked to significantly increased odds of MetS and central obesity among participants with insufficient physical activity.

This cross-sectional study revealed that the prevalence of childhood MetS in Guangzhou was 2.20%, lower than the national average of 4.0% in China (under the same diagnostic criteria) [28]. Compared with other cities in southern China, the rate is similar to that in Changzhou (2.3%) [47]. Overall, the prevalence of childhood MetS in Guangzhou is relatively low, which may be attributed to the city’s developed economy and local dietary structure [4,6]. Boys had a higher prevalence of MetS, central obesity, elevated blood pressure, and dyslipidemia than girls. This might be due to the rise in androgen levels during boys’ puberty, which can lead to more deep belly fat and activate the sympathetic nerve, both of which can increase the risk of cardiovascular diseases [48]. This study also revealed that increased screen time exhibited a positive correlation with central obesity and elevated blood pressure, which aligned with the findings of research conducted in Pakistan [49]. Furthermore, this study found that individuals with elevated blood pressure had shorter sleep durations, while those with hyperglycemia, hypertriglyceridemia, and dyslipidemia showed longer sleep durations. This seemingly contradictory phenomenon potentially reflects a U-shaped association between sleep duration and metabolic diseases [50]. Both too-short or too-long sleep duration can lead to metabolic disorders and increase the risk of illness.

This study was conducted using RRR analysis to derive a dietary pattern associated with SUA. The identified dietary pattern was characterized by high consumption of meat & meat products and beverages, and low consumption of fresh vegetables, fresh fruits & their products, eggs, dairy products, and sugary food. This pattern is consistent with previous studies linking such diets to elevated SUA levels. For example, a study on children and adolescents in Shenzhen reported that a meat-based dietary pattern was positively associated with SUA, while a vegetarian pattern was negatively associated [51]. Similarly, another study on Chinese adults using RRR found that the SUA-related dietary pattern was characterized by high intake of poultry, sugary drinks, and animal offal, alongside low intake of desserts and snacks, which is highly consistent with our study [44].

After accounting for potential confounding factors, higher adherence to the SUA-related dietary pattern was significantly associated with an increased likelihood of having MetS. This finding aligns with previous reports—yet not definitive evidence—suggesting that SUA might lie on a putative pathway linking diet to MetS. For example, both NHANES data and a Taiwanese adolescent study showed that SUA mediates the diet–MetS relationship, accounting for 25% of the diet quality effect on MetS risk in adults [52] and 31.1% of the mediation effect of fructose-rich beverage intake on MetS in adolescents [53]. However, these mediation analyses were cross-sectional and therefore remain hypothesis-generating. In terms of food composition, high intake of meat & meat products boosts purine intake (purines break down into uric acid), while high beverage intake may lead to excessive fructose, further elevating SUA levels [51,54]. Meanwhile, low intake of fresh fruits and vegetables reduces dietary fiber, potentially inhibiting uric acid excretion [55]. These pathways may exacerbate insulin resistance and chronic inflammation through sustained elevation of SUA—both key mechanisms in the pathogenesis of MetS [56,57]. Further longitudinal studies and experimental validation are warranted to elucidate its biological mechanisms and clinical utility.

Our research demonstrated that higher adherence to the SUA-related dietary pattern was linked to a higher likelihood of central obesity. This echoes a systematic review of prospective studies showing that ultra-processed food consumption, like beverages, was linked to greater weight gain and central adiposity [58]. Our prior study similarly confirmed that a rice-and-meat dietary pattern was linked to an increased prevalence of central obesity in children [29]. These findings point to a potential link between the SUA-related dietary pattern and visceral fat accumulation via elevated SUA and oxidative stress [59]. Plausibly, a bidirectional interaction exists between SUA and central obesity: on one hand, high fructose intake activates carbohydrate-responsive element-binding protein (ChREBP) and upregulates fatty acid synthase (FAS), promoting lipogenesis and fat deposition [60]; on the other hand, excess adipose tissue increases leptin secretion, which activates the renin–angiotensin–aldosterone system (RAAS) and impairs renal uric acid excretion [61].

In our study, subgroup analyses showed that in the insufficient physical activity group, higher adherence to the SUA-related dietary pattern was more strongly associated with increased odds of MetS and central obesity. This is consistent with findings from Spanish adults, which reported that individuals with the highest MetS severity scores engaged less in moderate-to-vigorous physical activity and had greater sedentary time [62]. Similarly, an Italian interventional study confirmed that high-intensity physical activity can enhance the effectiveness of a Mediterranean diet in improving MetS among adolescents [63]. These results imply that the combination of poor diet and low physical activity may significantly elevate MetS risk.

In this study, the SUA-related dietary pattern did not reveal significant associations with elevated blood pressure, hyperglycemia, hypertriglyceridemia, or dyslipidemia, suggesting that such correlations may not emerge until early adulthood [64]. This delayed manifestation might reflect age-dependent vascular adaptability—younger individuals preserve vascular elasticity, which might temporarily buffer dietary-induced hypertensive effects [65].

To our knowledge, this is the first study to utilize the RRR method to uncover the SUA-related dietary pattern and examine its links to MetS and its diagnostic components among Chinese children. However, several limitations should be acknowledged. Firstly, this cross-sectional design precludes causal inference. Mechanistic interpretations presented in Section 4 of this paper are therefore speculative and should be tested in prospective or interventional studies. Secondly, the research sample was from Guangzhou, and the unique dietary structure and cooking methods in the Lingnan region may limit the generalizability of this pattern. Moreover, this dietary pattern has not been independently verified in other samples. Thirdly, the FFQ did not distinguish between refined and whole-grain cereals, which limits our ability to interpret dietary quality and metabolic risk. Fourthly, dietary intake was self-reported via the FFQ; although trained interviewers provided assistance, the limited recall accuracy of children aged 9–12 years may have introduced reporting bias. Additionally, residual confounding cannot be ruled out; residual confounding by total energy intake may persist, and unmeasured factors such as household income and genetic variables could also confound associations.

## 5. Conclusions

In summary, an SUA-related dietary pattern was identified in Chinese children. Higher adherence to the SUA-related dietary pattern was associated with an elevated risk of MetS, particularly among those with insufficient physical activity. These findings have important clinical and public health implications for children. According to our results, dietary adjustments can reduce MetS risk in high-risk individuals. This involves optimizing the SUA-related dietary pattern by reducing the intake of certain foods (such as meat and beverages) and increasing the intake of others (such as fresh vegetables and fruits).

## Figures and Tables

**Table 1:** General characteristics of the study population with different metabolic syndrome status.

	Metabolic Syndrome		*Z/χ* ^2^	*p*-Value
	NO (*n* = 3837)	YES (*n* = 86)		
Age in years, M (P_25_, P_75_)	13.89 (11.73, 15.96)	13.83 (11.56, 15.88)	0.05	0.821
Sex, *n* (%)			11.65	**<0.001**
Male	2031 (97.04)	62 (2.96)		
Female	1806 (98.69)	24 (1.31)		
The education of father, *n* (%)			0.25	0.881
Junior high school and below	1138 (97.93)	24 (2.07)		
Senior high school	1201 (97.64)	29 (2.36)		
College degree or above	1498 (97.84)	33 (2.16)		
The education of mother, *n* (%)			2.47	0.291
Junior high school and below	1244 (98.34)	21 (1.66)		
Senior high school	1129 (97.58)	28 (2.42)		
College degree or above	1464 (97.53)	37 (2.47)		
Boarding, *n* (%)			0.79	0.374
Yes	2258 (98.00)	46 (2.00)		
No	1579 (97.53)	40 (2.47)		
Insufficient physical activity, *n* (%)			1.95	0.163
Yes	2186 (98.11)	42 (1.89)		
No	1651 (97.40)	44 (2.60)		
Screen time (min/d), M (P_25_, P_75_)	60.00 (34.29, 102.86)	68.57 (34.29, 102.86)	0.35	0.552
Sleep duration (h/d), M (P_25_, P_75_)	8.95 (8.17, 9.83)	8.90 (8.18, 10.03)	0.79	0.374
MetS components, M (P_25_, P_75_)				
Waist circumference (cm)	64.00 (59.20, 69.20)	85.90 (82.40, 90.68)	214.09	**<0.001**
Systolic blood pressure (mmHg)	110.50 (102.00, 118.00)	122.50 (114.00, 131.75)	81.31	**<0.001**
Diastolic blood pressure (mmHg)	67.00 (62.50, 72.00)	72.00 (68.00, 79.38)	44.49	**<0.001**
FBG (mmol/L)	4.91 (4.65, 5.16)	5.15 (4.93, 5.61)	40.55	**<0.001**
TG (mmol/L)	0.82 (0.65, 1.08)	1.68 (1.47, 2.11)	155.10	**<0.001**
HDL-C (mmol/L)	1.35 (1.18, 1.53)	1.13 (1.00, 1.29)	59.72	**<0.001**
non-HDL-C (mmol/L)	2.57 (2.17, 2.99)	3.43 (2.75, 3.96)	77.77	**<0.001**
SUA (mg/dL), M (P_25_, P_75_)	6.25 (5.34, 7.29)	8.09 (6.85, 9.02)	76.90	**<0.001**

The data are presented as the number of cases (%) for categorical variables and as medians with interquartile ranges [M (P_25_, P_75_)] for continuous variables. Statistical analyses were conducted using the Mann–Whitney U test and the chi-square trend test. A bolded *p*-value indicates significance at the level of less than 0.05.

**Table 2:** General characteristics of the study population with different status of individual MetS components.

	Central Obesity		*p*-Value	Elevated Blood Pressure		*p*-Value	Hyperglycemia		*p*-Value	Hypertriglyceridemia		*p*-Value	Dyslipidemia		*p*-Value
	NO (*n* = 3466)	YES (*n* = 457)		NO (*n* = 3481)	YES (*n* = 442)		NO (*n* = 3755)	YES (*n* = 168)		NO (*n* = 3551)	YES (*n* = 372)		NO (*n* = 3360)	YES (*n* = 603)	
Age in years, M (P_25_, P_75_)	13.87 (11.75, 15.94)	14.02 (11.60, 16.01)	0.589	13.81 (11.65, 15.92)	14.39 (12.77, 16.12)	**<0.001**	13.96 (11.79, 15.97)	12.76 (11.29, 14.13)	**<0.001**	13.90 (11.74, 15.95)	13.74 (11.61, 15.96)	0.738	13.94 (11.74, 15.96)	13.66 (11.69, 15.91)	0.172
Sex, *n* (%)			**<0.001**			**<0.001**			0.214			0.741			**<0.001**
Male	1803 (86.14)	290 (13.86)		1816 (86.77)	277 (13.23)		1995 (95.32)	98 (4.68)		1891 (90.35)	202 (9.65)		1752 (83.71)	341 (16.29)	
Female	1663 (90.87)	167 (9.13)		1665 (90.98)	165 (9.02)		1760 (96.17)	70 (3.83)		1660 (90.71)	170 (9.29)		1608 (87.87)	222 (12.13)	
The education of father, *n* (%)			0.164			0.939			0.181			**0.034**			0.189
Junior high school and below	1028 (88.47)	134 (11.53)		1028 (88.47)	134 (11.53)		1118 (96.21)	44 (3.79)		1039 (89.41)	123 (10.59)		977 (84.08)	185 (15.92)	
Senior high school	1102 (89.59)	128 (10.41)		1092 (88.78)	138 (11.22)		1183 (96.18)	47 (3.82)		1103 (89.67)	127 (10.33)		1063 (86.42)	167 (13.58)	
College degree or above	1336 (87.26)	195 (12.74)		1361 (88.90)	170 (11.10)		1454 (94.97)	77 (5.03)		1409 (92.03)	122 (7.97)		1320 (86.22)	211 (13.78)	
The education of mother, *n* (%)			0.059			0.373			0.282			0.057			0.944
Junior high school and below	1140 (90.12)	125 (9.88)		1121 (88.62)	144 (11.38)		1219 (96.36)	46 (3.64)		1134 (89.64)	131 (10.36)		1085 (85.77)	180 (14.23)	
Senior high school	1013 (87.55)	144 (12.45)		1016 (87.81)	141 (12.19)		1108 (95.76)	49 (4.24)		1037 (89.63)	120 (10.37)		993 (85.83)	164 (14.17)	
College degree or above	1313 (87.48)	188 (12.52)		1344 (89.54)	157 (10.46)		1428 (95.14)	73 (4.86)		1380 (91.94)	121 (8.06)		1282 (85.41)	219 (14.59)	
Boarding, *n* (%)			0.848			0.407			**<0.001**			0.660			**0.010**
Yes	2038 (88.45)	266 (11.55)		2053 (89.11)	251 (10.89)		2232 (96.88)	72 (3.12)		2090 (90.71)	214 (9.29)		1945 (84.42)	359 (15.58)	
No	1428 (88.20)	191 (11.80)		1428 (88.20)	191 (11.80)		1523 (94.07)	96 (5.93)		1461 (90.24)	158 (9.76)		1415 (87.40)	204 (12.60)	
Insufficient physical activity, *n* (%)			0.267			0.385			0.156			0.282			0.505
Yes	1980 (88.87)	248 (11.13)		1986 (89.14)	242 (10.86)		2142 (96.14)	86 (3.86)		2027 (90.98)	201 (9.02)		1916 (86.00)	312 (14.00)	
No	1486 (87.67)	209 (12.33)		1495 (88.20)	200 (11.80)		1613 (95.16)	82 (4.84)		1524 (89.91)	171 (10.09)		1444 (85.19)	251 (14.81)	
Screen time (min/d), M (P_25_, P_75_)	60.00 (34.29, 102.86)	68.57 (34.29, 107.14)	**0.009**	60.00 (34.29, 102.86)	68.57 (34.29, 111.43)	**0.032**	60.00 (34.29, 102.86)	51.43 (25.71, 102.86)	0.087	60.00 (34.29, 102.86)	56.43 (34.29, 101.79)	0.327	60.00 (34.29, 102.86)	60.00 (34.29, 102.86)	0.803
Sleep duration (h/d), M (P_25_, P_75_)	8.96 (8.17, 9.83)	8.93 (8.14, 9.75)	0.440	9.00 (8.17, 9.85)	8.76 (8.17, 9.62)	**0.011**	8.93 (8.17, 9.79)	9.49 (8.52, 10.33)	**<0.001**	8.93 (8.17, 9.79)	9.08 (8.33, 10.00)	**0.009**	8.93 (8.17, 9.80)	9.07 (8.29, 9.95)	**0.006**
SUA (mg/dL), M (P_25_, P_75_)	6.16 (5.29, 7.14)	7.14 (6.20, 8.55)	**<0.001**	6.20 (5.31, 7.22)	6.97 (5.98, 8.11)	**<0.001**	6.28 (5.36, 7.34)	6.07 (5.30, 7.15)	0.352	6.23 (5.33, 7.27)	6.65 (5.59, 8.10)	**<0.001**	6.25 (5.32, 7.26)	6.47 (5.52, 7.85)	**<0.001**

The data are presented as the number of cases (%) for categorical variables and as medians with interquartile ranges [M (P_25_, P_75_)] for continuous variables. Statistical analyses were conducted using the Mann–Whitney U test and the chi-square trend test. A bolded *p*-value indicates significance at the level of less than 0.05.

**Table 3:** General characteristics of the study population according to the tertiles of the SUA-related dietary pattern scores.

	SUA-Related Dietary Pattern Scores			*H/* *χ* ^2^	*p*-Value
	T1 (*n* = 1308)	T2 (*n* = 1308)	T3 (*n* = 1307)		
Age in years, M (P_25_, P_75_)	13.16 (11.21, 14.57)	13.96 (11.84, 15.96)	14.90 (12.97, 16.18)	224.33	**<0.001**
Sex, *n* (%)				146.65	**<0.001**
Male	539 (25.75)	707 (33.78)	847 (40.47)		
Female	769 (42.02)	601 (32.84)	460 (25.14)		
The education of father, *n* (%)				4.73	0.317
Junior high school and below	369 (31.76)	383 (32.96)	410 (35.28)		
Senior high school	433 (35.20)	402 (32.68)	395 (32.12)		
College degree or above	506 (33.05)	523 (34.16)	502 (32.79)		
The education of mother, *n* (%)				3.33	0.504
Junior high school and below	402 (31.78)	426 (33.68)	437 (34.54)		
Senior high school	386 (33.36)	396 (34.23)	375 (32.41)		
College degree or above	520 (34.64)	486 (32.38)	495 (32.98)		
Boarding, *n* (%)				106.12	**<0.001**
Yes	629 (27.30)	790 (34.29)	885 (38.41)		
No	679 (41.94)	518 (32.00)	422 (26.06)		
Insufficient physical activity, *n* (%)				1.66	0.436
Yes	725 (32.54)	757 (33.98)	746 (33.48)		
No	583 (34.40)	551 (32.51)	561 (33.09)		
Screen time (min/d), M (P_25_, P_75_)	51.43 (25.71, 94.29)	64.29 (34.29, 102.86)	68.57 (38.57, 115.71)	90.61	**<0.001**
Sleep duration (h/d), M (P_25_, P_75_)	9.25 (8.43, 10.11)	9.00 (8.25, 9.79)	8.58 (8.00, 9.46)	160.06	**<0.001**

The data are presented as the number of cases (%) for categorical variables and as medians with interquartile ranges [M (P_25_, P_75_)] for continuous variables. Statistical analyses were conducted using the Mann–Whitney U test and the chi-square trend test. A bolded *p*-value indicates significance at the level of less than 0.05.

**Table 4:** Association of the SUA-related dietary pattern scores with MetS prevalence and its individual diagnostic components.

SUA-Related Dietary Pattern Scores	Model 1 ^a^		Model 2 ^b^	
	OR (95% CI)	*p*-Value	OR (95% CI)	*p*-Value
Metabolic Syndrome				
T1	1.00	—	1.00	—
T2	1.36 (0.79–2.39)	0.266	1.31 (0.75–2.31)	0.344
T3	1.55 (0.91–2.68)	0.106	1.43 (0.82–2.55)	0.211
Linear for 1 unit	1.30 (1.04–1.64)	**0.022**	1.27 (1.00–1.62)	**0.049**
Central Obesity				
T1	1.00	—	1.00	—
T2	1.56 (1.21–2.02)	**<0.001**	1.50 (1.16–1.96)	**0.002**
T3	1.87 (1.46–2.40)	**<0.001**	1.72 (1.33–2.25)	**<0.001**
Linear for 1 unit	1.29 (1.16–1.43)	**<0.001**	1.24 (1.11–1.38)	**<0.001**
Elevated Blood Pressure				
T1	1.00	—	1.00	—
T2	1.45 (1.13–1.87)	**0.003**	1.31 (1.02–1.70)	**0.036**
T3	1.36 (1.06–1.76)	**0.016**	1.12 (0.85–1.46)	0.420
Linear for 1 unit	1.12 (1.01–1.24)	**0.027**	1.03 (0.92–1.15)	0.634
Hyperglycemia				
T1	1.00	—	1.00	—
T2	0.64 (0.44–0.93)	**0.021**	0.71 (0.48–1.03)	0.070
T3	0.63 (0.43–0.91)	**0.016**	0.76 (0.51–1.12)	0.171
Linear for 1 unit	0.83 (0.72–0.97)	**0.014**	0.90 (0.77–1.06)	0.193
Hypertriglyceridemia				
T1	1.00	—	1.00	—
T2	1.19 (0.92–1.54)	0.189	1.22 (0.93–1.59)	0.146
T3	0.98 (0.75–1.29)	0.896	1.03 (0.77–1.37)	0.845
Linear for 1 unit	1.02 (0.91–1.13)	0.785	1.04 (0.92–1.17)	0.541
Dyslipidemia				
T1	1.00	—	1.00	—
T2	1.12 (0.90–1.40)	0.293	1.09 (0.88–1.37)	0.432
T3	1.01 (0.81–1.26)	0.949	0.97 (0.76–1.22)	0.779
Linear for 1 unit	1.00 (0.92–1.10)	0.939	0.98 (0.89–1.09)	0.758

OR, odds ratio; CI, confidence interval. ^a^ Model 1: crude; ^b^ Model 2: adjusted for age, sex, boarding status, insufficient physical activity, screen time, sleep duration, the education level of father and mother. The bold *p*-value means “<0.05”.

**Table 5:** Association of the SUA-related dietary pattern scores with MetS and its individual diagnostic components in children of different physical activity sufficiency.

	SUA-Related Dietary Pattern Scores						
	T1	T2		T3		Linear for 1 unit	
		OR (95% CI)	*p*-value	OR (95% CI)	*p*-value	OR (95% CI)	*p*-value
Insufficient physical activity group	—	—		—		—	
Metabolic Syndrome	1.00	3.07 (1.23–8.92)	**0.015**	3.74 (1.49–10.89)	**0.004**	1.69 (1.18–2.42)	**0.004**
Central Obesity	1.00	2.28 (1.57–3.38)	**<0.001**	2.64 (1.80–3.92)	**<0.001**	1.39 (1.20–1.62)	**<0.001**
Elevated Blood Pressure	1.00	1.29 (0.91–1.84)	0.155	1.23 (0.86–1.77)	0.268	1.07 (0.92–1.24)	0.396
Hyperglycemia	1.00	0.72 (0.43–1.20)	0.213	0.61 (0.34–1.07)	0.087	0.83 (0.68–1.03)	0.089
Hypertriglyceridemia	1.00	1.44 (0.99–2.11)	0.058	1.47 (0.99–2.18)	0.058	1.17 (0.99–2.11)	0.060
Dyslipidemia	1.00	0.88 (0.65–1.18)	0.370	0.88 (0.65–1.21)	0.442	1.00 (0.88–1.14)	0.991
Sufficient physical activity group	—	—		—		—	
Metabolic Syndrome	1.00	0.69 (0.32–1.48)	0.325	0.69 (0.32–1.48)	0.338	0.97 (0.70–1.36)	0.870
Central Obesity	1.00	0.97 (0.67–1.42)	0.894	1.13 (0.78–1.64)	0.516	1.09 (0.93–1.28)	0.315
Elevated Blood Pressure	1.00	1.32 (0.91–1.93)	0.141	0.98 (0.66–1.46)	0.914	0.97 (0.83–1.14)	0.721
Hyperglycemia	1.00	0.67 (0.37–1.18)	0.170	0.96 (0.55–1.67)	0.899	0.99 (0.79–1.26)	0.949
Hypertriglyceridemia	1.00	1.03 (0.71–1.50)	0.877	0.67 (0.43–1.02)	0.062	0.91 (0.77–1.07)	0.246
Dyslipidemia	1.00	1.41 (1.01–1.97)	**0.047**	1.05 (0.73–1.51)	0.778	0.95 (0.82–1.10)	0.499

OR, odds ratio; CI, confidence interval. All models were adjusted for age, sex, boarding status, screen time, sleep duration, and the education level of father and mother. A bolded *p*-value indicates significance at the level of less than 0.05.

## Data Availability

The datasets generated and/or analyzed during the current study are not publicly available due to privacy but are available from the corresponding authors on reasonable request.

## Supplementary Materials

The following supporting information can be downloaded at: https://www.mdpi.com/article/10.3390/nu17162618/s1, Table S1: Food groups used in the reduced-rank regression analysis; Table S2: Factor loading of 15 food groups in the SUA-related dietary pattern derived using reduced-rank regression (RRR) among the study population.

## Abbreviations

The following abbreviations are used in this manuscript:

MetS	Metabolic syndrome
SUA	Serum uric acid
RRR	Reduced-rank regression
DASH	Dietary Approaches to Stop Hypertension
FFQ	Food frequency questionnaire
FBG	Blood glucose
TG	Triglycerides
TC	Total cholesterol
HDL-C	High-density lipoprotein cholesterol
non-HDL-C	Non-high-density lipoprotein cholesterol
P_90_	The 90th percentile
P_95_	The 95th percentile
OR	Odds ratio
CI	Confidence interval
ChREBP	Carbohydrate-responsive element-binding protein
FAS	Fatty acid synthase
RAAS	Renin–angiotensin–aldosterone system
