# Supplementary material for: Association of a Serum Uric Acid-Related Dietary Pattern with Metabolic Syndrome Among Guangzhou Children Aged 9–17 Years: A Cross-Sectional Study
Source: Nutrients. 2025 Aug 13;17(16):2618. doi: 10.3390/nu17162618 (PMC12389420; doi:10.3390/nu17162618)
Supplement: Supplementary file 1 [file nutrients-17-02618-s001.zip › nutrients-3775925-supplementary.pdf]

Table S1. Food groups used in the reduced rank regression analysis.

Number	Food Group	Examples of food items
1	Cereal & tuber crops	rice and its products, wheat and its products, corn and its products, potato and its products
2	Beans & its products	Soybean, soybean milk, tofu, bean curd, dried bean curd
3	Fresh vegetables	Cabbage, tomato, lettuce
4	Pickled vegetables	Pickled mustard greens, sour pickles
5	Mushrooms & algae	Mushroom, laver, kelp
6	Fresh fruits & their products	Bananas, apples, berries, dried fruits
7	Dairy products	Milk, milk powder, yogurt, cheese
8	Meat & meat products	Pork, beef, goat, lamb, chicken, duck, goose, animal organ (liver, heart, kidney, lung, etc.), ham sausage, bacon
9	Aquatic products	Fish, shrimp, crab
10	Eggs	Eggs
11	Nuts	Peanuts, almonds, walnuts, hazelnuts
12	Sugary food	Honey, jam, jelly, candies, candied fruit
13	Convenience food	Instant noodles, instant rice noodles, cookies, cakes, bread, spicy strips, fried puffed snacks.
14	Fast food	Hamburger, fried chicken, French fries
15	Beverages	Carbonated drinks, prepackaged juice, milk beverages, sodas, milky tea, sweet tea beverages, vegetable protein drinks, sports beverages, ice cream

Table S2. Factor loading of 15 food groups in the SUA-related dietary pattern derived using reduced rank regression (RRR) among the study population.

Number	Food Group	Factor loadings
1	Cereal & tuber crops	–0.09
2	Beans & its products	0.11
3	Fresh vegetables	–0.27
4	Pickled vegetables	–0.10
5	Mushrooms & algae	–0.17
6	Fresh fruits & their products	–0.54
7	Dairy products	–0.27
8	Meat & meat products	0.45
9	Aquatic products	–0.01
10	Eggs	–0.23
11	Nuts	–0.11
12	Sugary food	–0.40
13	Convenience food	0.04
14	Fast food	0.15
15	Beverages	0.23
